# Using Local Convolutional Neural Networks for Genomic Prediction

**DOI:** 10.3389/fgene.2020.561497

**Published:** 2020-11-12

**Authors:** Torsten Pook, Jan Freudenthal, Arthur Korte, Henner Simianer

**Affiliations:** ^1^Animal Breeding and Genetics Group, Department of Animal Sciences, Center for Integrated Breeding Research, University of Goettingen, Göttingen, Germany; ^2^Center for Computational and Theoretical Biology, University of Wuerzburg, Wuerzburg, Germany

**Keywords:** phenotype prediction, Keras, genomic selection, selection, breeding, machine learning, deep learning

## Abstract

The prediction of breeding values and phenotypes is of central importance for both livestock and crop breeding. In this study, we analyze the use of artificial neural networks (ANN) and, in particular, local convolutional neural networks (LCNN) for genomic prediction, as a region-specific filter corresponds much better with our prior genetic knowledge on the genetic architecture of traits than traditional convolutional neural networks. Model performances are evaluated on a simulated maize data panel (*n* = 10,000; *p* = 34,595) and real Arabidopsis data (*n* = 2,039; *p* = 180,000) for a variety of traits based on their predictive ability. The baseline LCNN, containing one local convolutional layer (kernel size: 10) and two fully connected layers with 64 nodes each, is outperforming commonly proposed ANNs (multi layer perceptrons and convolutional neural networks) for basically all considered traits. For traits with high heritability and large training population as present in the simulated data, LCNN are even outperforming state-of-the-art methods like genomic best linear unbiased prediction (GBLUP), Bayesian models and extended GBLUP, indicated by an increase in predictive ability of up to 24%. However, for small training populations, these state-of-the-art methods outperform all considered ANNs. Nevertheless, the LCNN still outperforms all other considered ANNs by around 10%. Minor improvements to the tested baseline network architecture of the LCNN were obtained by increasing the kernel size and of reducing the stride, whereas the number of subsequent fully connected layers and their node sizes had neglectable impact. Although gains in predictive ability were obtained for large scale data sets by using LCNNs, the practical use of ANNs comes with additional problems, such as the need of genotyping all considered individuals, the lack of estimation of heritability and reliability. Furthermore, breeding values are additive by design, whereas ANN-based estimates are not. However, ANNs also comes with new opportunities, as networks can easily be extended to account for additional inputs (omics, weather etc.) and outputs (multi-trait models), and computing time increases linearly with the number of individuals. With advances in high-throughput phenotyping and cheaper genotyping, ANNs can become a valid alternative for genomic prediction.

## 1. Introduction

The prediction of breeding values and phenotypes is of central importance for both livestock and crop breeding. Obtaining accurate estimates of breeding values at an earlier stage can impact the decision on which individuals should remain in a breeding programs, shorten the generation interval and thus lead to higher genetic gains per year (Schaeffer, 2006). Because of this, optimizing breeding schemes is of key importance for overcoming the global challenges of feeding a planet with a growing Human population (Foley et al., 2011).

With the increasing and cheaper availability of genomic data, the estimation of genomic breeding values has become an important part of breeding. Over the years, a variety of methods for the prediction of breeding values and phenotypes have been proposed with the most commonly applied methods being based on linear mixed models (genomic best linear unbiased prediction; GBLUP) and Bayesian linear models (BayesA, BayesB, BayesC, Bayesian Lasso) (Meuwissen et al., 2001; Gianola et al., 2009). Currently, variations of these approaches have been successfully implemented in both livestock (Hayes et al., 2009; Hayes and Goddard, 2010; Gianola and Rosa, 2015) and plant breeding (Jannink et al., 2010; Albrecht et al., 2011; Nakaya and Isobe, 2012; Heslot et al., 2015). Since breeding values are additive by design, most of these models only account for additive single marker effects, but extension to account for dominance and epistatic interactions have been proposed (Da et al., 2014; Jiang and Reif, 2015; Martini et al., 2017) and are regularly applied for the prediction of phenotypes.

The computational load, both in terms of computing time and memory requirements are common problems when performing genomic prediction on large scale data set, as computing time in the mixed model is increasing cubically in the number of phenotyped individuals considered. In particular for large scale livestock populations approximations such as the proven-young algorithm (Misztal et al., 2014) for the inversion of the genomic relationship matrix have been proposed to reduce the computational load. Thus, enabling the use of genetic evaluations in large scale genetic evaluations such as the US Holstein population with 569,404 genotyped animals (Masuda et al., 2016). Similarly, computing time and memory requirements tend to explode in state-of-the-art methods such as GBLUP when additional input dimensions like weather data (Gillberg et al., 2019) are considered or multi-trait models are used.

**Figure 1:**
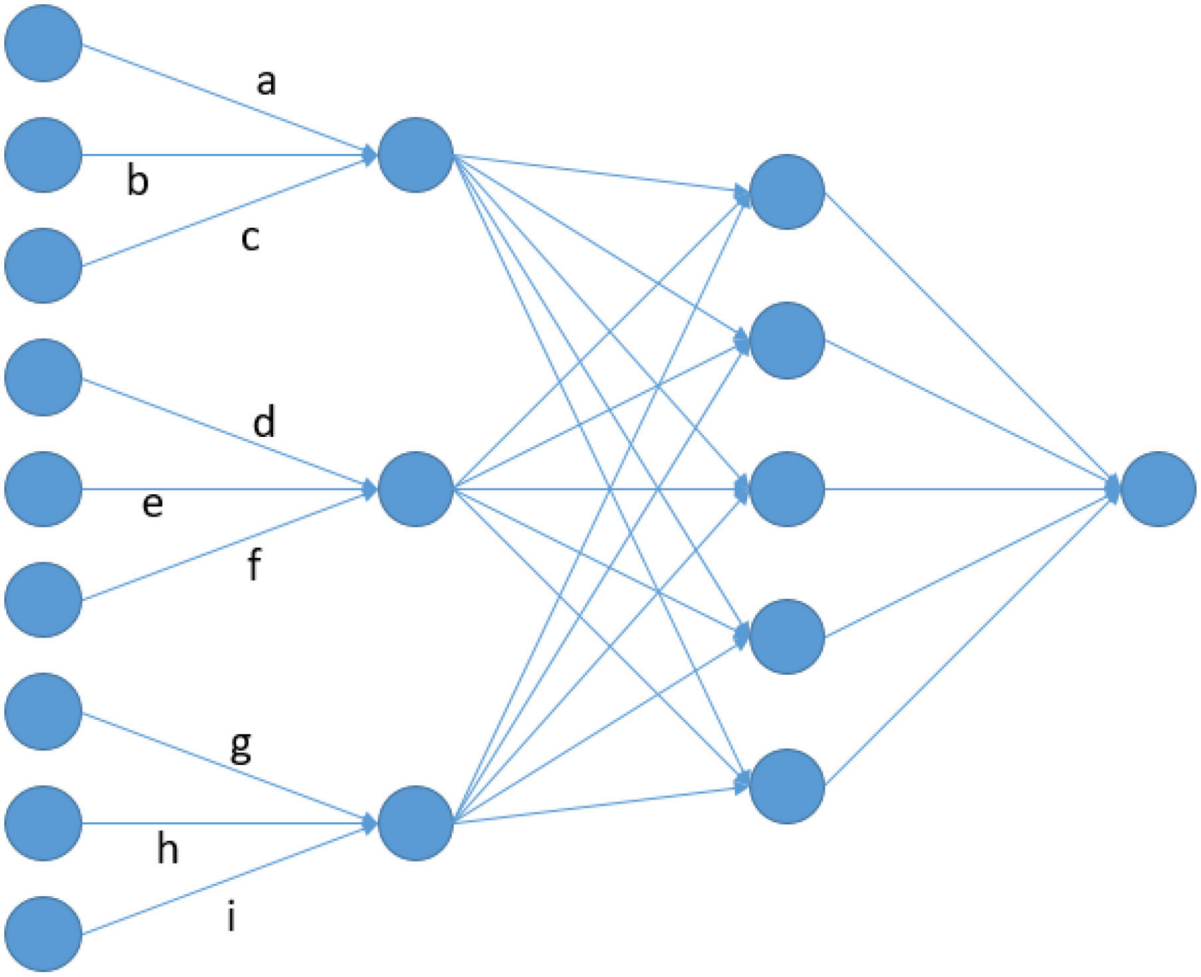
Node architecture of a LCNN containing a LCL with window size and stride of 3 and a FCL with 5 nodes.

With increasing computational power, more and more researchers have started using Deep Learning methods and, in particular, artificial neural networks (ANN) in genetics (Eraslan et al., 2019). As a results of this, a series of studies have recently been carried out to analyse the use of ANNs for genomic prediction (Bellot et al., 2018; Ma et al., 2018; Waldmann, 2018; Azodi et al., 2019; Khaki and Wang, 2019; Montesinos-López et al., 2019; Pérez-Enciso and Zingaretti, 2019; Abdollahi-Arpanahi et al., 2020). However, the common result in those studies is that state-of-the-art methods such as GBLUP or methods from the Bayesian alphabet (Meuwissen et al., 2001; Gianola et al., 2009) have a similar or even better performance. In cases for which improvements were obtained, either very specific trait architectures are considered (Waldmann, 2018), improvements are not consistent across traits (Bellot et al., 2018; Montesinos-López et al., 2019) or additional data like environmental information is used (Khaki and Wang, 2019). Note that for most traits considered in those studies, the best performing ANNs are usually multi-layer-perceptrons (MLP) with one or sometimes two fully-connected layers (FCL) between input and output layers (Bellot et al., 2018; Montesinos-López et al., 2019). Performance obtained with convolutional neural networks (CNN) are usually similar or even slightly worse (Bellot et al., 2018) with best performing models using filters with very small kernels. Here, kernel is referring to the number of adjacent markers considered in a single filter/convolution (Figure 1).

The key idea of convolutional layers (CL) is that effects are assigned to specific sequences of alleles. However, the same sequence of marker variants in different areas of the genome can have totally different effects as adjacent variants on a genotyping array have no direct functional relation (e.g., protein coding). Therefore, CNNs do not seem to be an appropriate network architecture for genomic prediction via array data. In this manuscript, we are proposing the use of local convolutional layers (LCL), as they provide a natural extension to CLs that fixes these issues by applying a region specific filter. As a toy example consider the artificial neural network given in Figure 1. In a CL *a, d, g* would be set to be equal, leading to the same filter being applied across the whole input. On the contrary, those parameters are fitted independently from each other in a LCL. This leads to a model with more parameters, but still far less than a MLP, where all inputs [here: single-nucleotide polymorphisms (SNP)] would be linked to all nodes of the first layer. For example, a FCL with 64 nodes would result in about 64 times more parameters than a LCL between input and the first layer. Furthermore, a CL layer with a filter linking 10 markers would only lead to 11 parameter (10 + intercept) while in a LCL the number of parameters would be slightly higher than the number of SNPs. As subsequent layer will typically have far more parameters, differences in this layer should still be neglectable. The aim here is to analyze the usefulness of the LCNN compared to traditional network architectures and state-of-the-art methods for a variety of trait architectures, heritabilities and population sizes. Furthermore, the ideal network architectures of a LCNN is investigated by performing benchmark tests with varying kernel sizes in the LCL and layer designs in subsequent FCLs. All tests were conducted on a simulated large scale data set in maize and a variety of smaller real *Arabidopsis thaliana* data panels.

## 2. Materials and Methods

### 2.1. Material

As a first data set, a simulated data panel containing 10,000 maize lines that were genotyped at 34,595 SNPs was generated. This was done by simulating random matings between 235 founder lines, with genotypes for the founder lines being generated by combining two haplotypes from a set of 470 doubled haploid lines of the European maize landrace Kemater Landmais Gelb for each founder. Simulations were executed in the software MoBPS (Pook et al., 2020) with the source code for the simulation given in Supplementary File 1. Founder lines were genotyped via the Affymetrix Axiom Maize Genotyping Array (Unterseer et al., 2014) and reduced via LD pruning in PLINK (Purcell et al., 2007). The interested reader is referred to Hölker et al. (2019) for details on the data generation procedure of the doubled haploid lines. Furthermore, a variety of traits were simulated with two main types of quantitative trait loci: Additive single marker QTL, assigning a linear effect to a single marker, and epistatic QTLs that are caused by the interaction between two genomic markers with nine separate effects for all marker combinations (00, 01,…,22). For the epistatic QTLs, we additionally considered cases where the two involved markers were physically linked. We will report results for six traits with the following architectures:

- 10 additive QTLs
- 1,000 additive QTLs
- 10 non-linked epistatic QTLs
- 1,000 non-linked epistatic QTLs
- 10 linked epistatic QTLs (QTL-markers being at most 5 SNPs apart)
- 1,000 linked epistatic QTLs (QTL-markers being at most 5 SNPs apart).

Individual QTL effect sizes were either assumed to be all the same or drawn from either a gaussian or gamma distribution, as true underlying effect structures in practice are not well known and can be highly dependent on the trait of interest. Since results were similar for the different distributions used to generate the individual QTL effect sizes, we will only report results for the tests conducted with equal QTL effect size for the additive QTLs and effects drawn from a gaussian distribution for the epistatic QTLs. For each trait, residual variances were varied to obtain traits with a heritability *h*^2^ of 0.1, 0.3, 0.5, 0.8, and 1. Furthermore, a set of 5 correlated traits with 1,000 underlying additive QTLs each was simulated by the use of *shuffle.trait()* in MoBPS (Pook et al., 2020). Correlation between traits was set to be between 0.0 and 0.8 and effect sizes were drawn from a gaussian distribution. Details on the generation of correlated traits in MoBPS can be found in the MoBPS guidelines (available at https://github.com/tpook92/MoBPS). Source code for the generation of all simulated traits can be found in Supplementary File 1.

Overall, this resulted in a data set of highly related individuals, as commonly present in breeding application and covered a variety of trait architectures ranging from relatively simple traits with purely additive traits caused by a few markers to highly complex traits caused by epistatic interactions between a high number of involved QTLs. It should be noted that all individuals considered in subsequent tests are part of the same generation. Since population structure can vary heavily depending on the species and the applied prediction methods can become more complex to include parental effects or similar, this was neglected there. Likewise, no additional fixed effects were simulated. In principle, these two factors could for example be accounted for by additional input layers and one-hot-encoded variables for the respective effects.

As a second data set, a real data panel from the 1,001 genomes project of *Arabidopsis thaliana* (Alonso-Blanco et al., 2016) was considered. After quality control, filtering for minor allele frequency and LD pruning, we reduced the available 10.7 M SNPs to 180k SNPs for 2,029 lines. Tests were conducted for 50 different traits, with between 83 and 468 phenotyped lines for each respective trait (Atwell et al., 2010; Li et al., 2010; Meijón et al., 2014; Strauch et al., 2015; Seren et al., 2016). The interested reader is referred to Freudenthal (2020) for details on the data preparation steps. Note that lines in this panel should be far less related than in the simulated maize data set.

All ANNs were fitted using Keras (Chollet, 2015) with respective scripts being available in Supplementary Files 2, 3. The R-package rrBLUP (Endelman, 2011) was used for fitting of the GBLUP and extended GBLUP (EGBLUP) model. The R-package BGLR (Pérez and de los Campos, 2014) was used for training of all considered Bayesian models. Multi-trait GBLUP models were fitted using ASRemlR (Butler et al., 2009). Exemplary scripts used for the fitting of the GBLUP, EGBLUP, and all Bayesian models can be found in Supplementary File 4.

### 2.2. Design of the Neural Network

For all tested ANNs, the SNP data set with genotypes encoded as 0,1,2 was used as the input layer and centered phenotypes (*ȳ* = 0) as the output layer. In genomic prediction, and especially when using an ANN, the number of parameters is usually substantially higher than the number of individuals that can be used for the model fitting. Thus, leading to n < < p problems (Fan et al., 2014). In this study, we will compare four main classes of models:

1. Linear models (LM)
2. Multi-layer perceptrons (MLP)
3. Convolutional neural networks (CNN)
4. Local convolutional neural networks (LCNN).

We classify all models that assign linear effects to single parameters as linear models. As a baseline for the class LM, we are considering the GBLUP (Meuwissen et al., 2001) model with a genomic relationship matrix as proposed by VanRaden (2008). Furthermore, methods from the Bayesian alphabet (Gianola et al., 2009; de los Campos et al., 2013), that perform Bayesian linear regression with prior assumptions on the individual marker variance, are considered. As results for all Bayesian methods were similar, we will only present results of BayesA (marginal prior: scaled-t-distribution) in the manuscript. The interested reader is referred to Supplementary Table 5, for results when using BayesB, BayesC, Bayesian Lasso. As an example for a linear model that accounts for epistiatic interactions, we will also consider the EGBLUP model (Martini et al., 2017) that is designed to assign linear effects to specific marker combinations.

The other three classes considered describe different types of ANNs. Here, we define the class of MLPs as ANNs that only contain FCLs. In CNNs/LCNNs we are using an additional single CL/LCL in front of the FCLs without use of pooling. For all three ANN classes we tested different layer designs ranging from zero up to three FCLs with varying number of nodes. For the CNN and LCNN we also tested different designs for the convolutional layer, by adjusting the kernel size between 3 and 40 and/or reduction of the stride to allow for filters to be applied on overlapping windows. For all models the rectified linear unit activation function (ReLU) was used with an adaptive estimates of lower-order moments (Adam) optimizer (Kingma and Ba, 2014) to minimize the mean squared errors and a dropout rate of 0.3 after each layer (Chollet, 2015; Goodfellow et al., 2016) was used. Changes to activation function, optimizer, dropout rate and target function were also tested but only had minor effects and therefore are neglected in the following. The interested reader is referred to Freudenthal (2020) for detailed results on adaption on these parameters.

Models are compared based on their predictive ability, which we define as the correlation between the estimated breeding values and phenotypes of individuals that were not used for the model training. Unless otherwise mentioned, 80% of the samples are used for training and the remaining 20% for testing (test set). For example, this results in 8,000 individuals being used for model fitting in the maize data, whereas the size of the training panel for the Arabidopsis data will be trait specific.

### 2.3. Size and Structure of the Training Data

A well-known problem of ANNs is that over-fitting can occur after a high number of training epochs (Goodfellow et al., 2016). Therefore, we split the 8,000 samples used for model fitting for the simulated maize data into 7,000 samples used for the actual training of the model (training set) and 1,000 samples that are only used to determine at which state training should be stopped (validation set). After each epoch, the predictive ability of the model was derived based on the validation set and the best performing model from up to 50 epochs was used as the final model. In the same way, the validation set could also be used to derive the ideal network architecture of the ANN (Freudenthal, 2020).

To further investigate the impact of the size of the training population, we considered varying number of individuals for model fitting (100, 250, 500, 1,000, 2,000, 3,000, 4,000, 6,000, 8,000). The size of the validation set was adapted based on the size of the data panel (20, 50, 100, 200, 300, 400, 500, 750, 1,000). Note that the relative size of the validation set is higher for smaller data sets as a higher impact of the validation set was observed in such chases. The remaining individuals were all used as part of the test set.

As the size of the training panel was already extremely small for the Arabidopsis data, a second study was conducted in which no validation set was used, but instead a fixed number of 25 training epochs was performed, therefore not requiring a validation set.

All tests for the simulated maize data/Arabidopsis data were repeated 25/100 times respectively, with randomly sampled training, validation and test sets.

## 3. Results

### 3.1. Comparison Between Model Types

In the following, we will report results for a baseline model from each of the three ANN class:

1. MPL: 2 FCL with 64 nodes
2. CNN: CL with kernel size and stride 10 + 2 FCL with 64 nodes
3. LCNN: LCL with kernel size and stride 10 + 2 FCL with 64 nodes.

Minor improvements were obtained by tweaking parameter settings for selected traits but overall tendencies of predictive ability across kernel size and number of layers as well as nodes per layer were stable. More details on differences will be provided for the LCNN at the end of the results section. There was no clear best model from the LM class. We will consider GBLUP as the baseline, but also report results for BayesA (Meuwissen et al., 2001) and the EGBLUP model (Martini et al., 2017).

### 3.2. Simulated Data

Unless otherwise stated, all results reported are based on the traits with a simulated heritability of 0.5 and 8,000 individuals used for model training.

**Figure 2:**
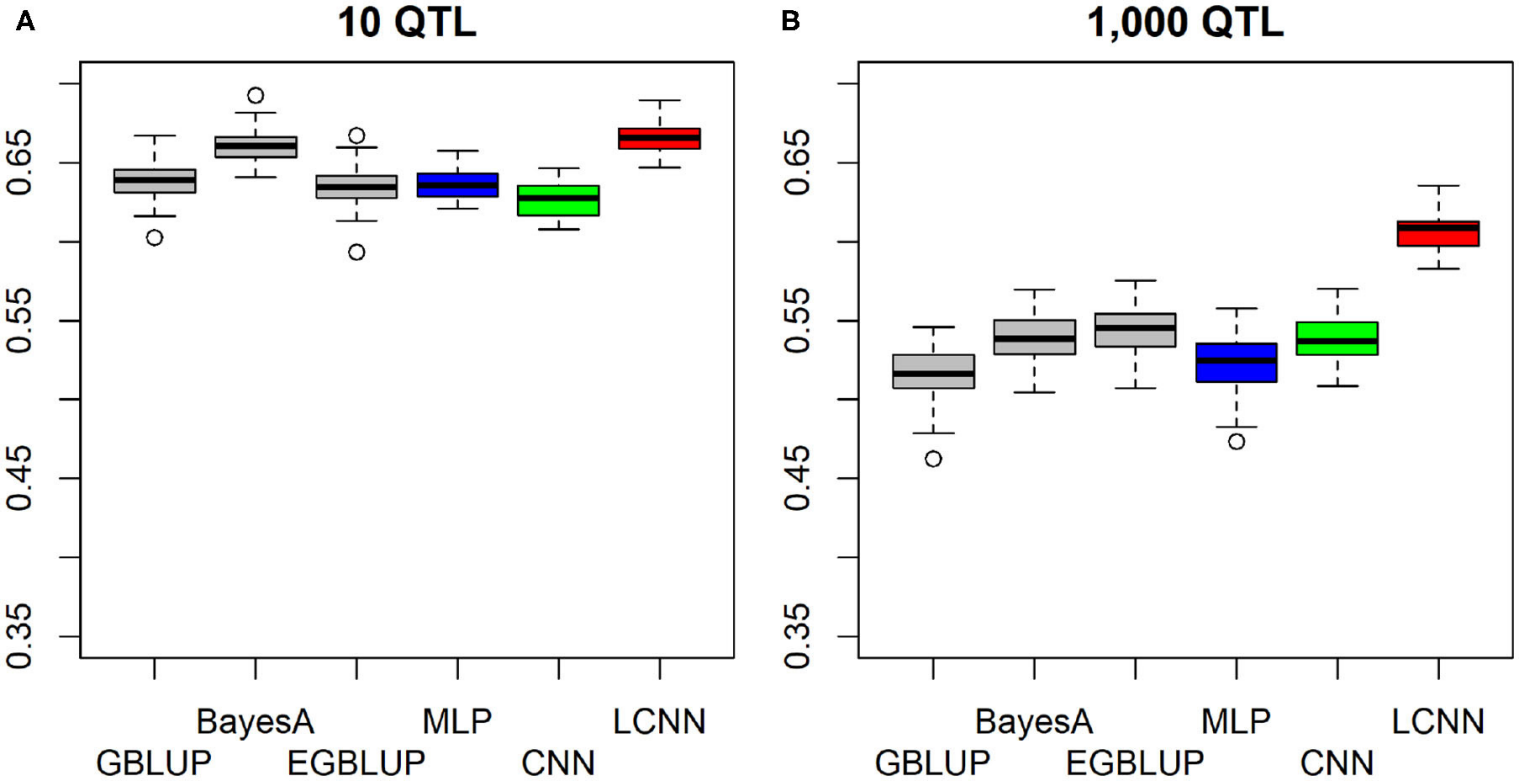
Predictive ability of different methods for genomic prediction for a simulated trait with 10 **(A)** and 1,000 **(B)** purely additive QTLs and a heritability of 0.5.

In the purely additive setting with just 10 underlying QTLs the highest predictive ability was obtained with the LCNN (0.666), outperforming the other three baseline models by around 0.03–0.04 (Figure 2A). When increasing the number of QTLs to 1,000, differences between LCNN (0.606) and the other three baseline models increased to around 0.06–0.09 (Figure 2B). The BayesA model led to similar predictive ability (0.660) as the LCNN for 10 QTLs but was outperformed (0.538) in case of 1,000 underlying QTLs. Even though the simulated traits had a purely additive genetic background, the EGBLUP model led to very similar or even slightly higher predictive ability than the GBLUP model. A potential reason for this could be “phantom epistatis” (de los Campos et al., 2019) as the use of pair-wise marker interactions could lead to a better overall representation of haplotype similarities.

**Figure 3:**
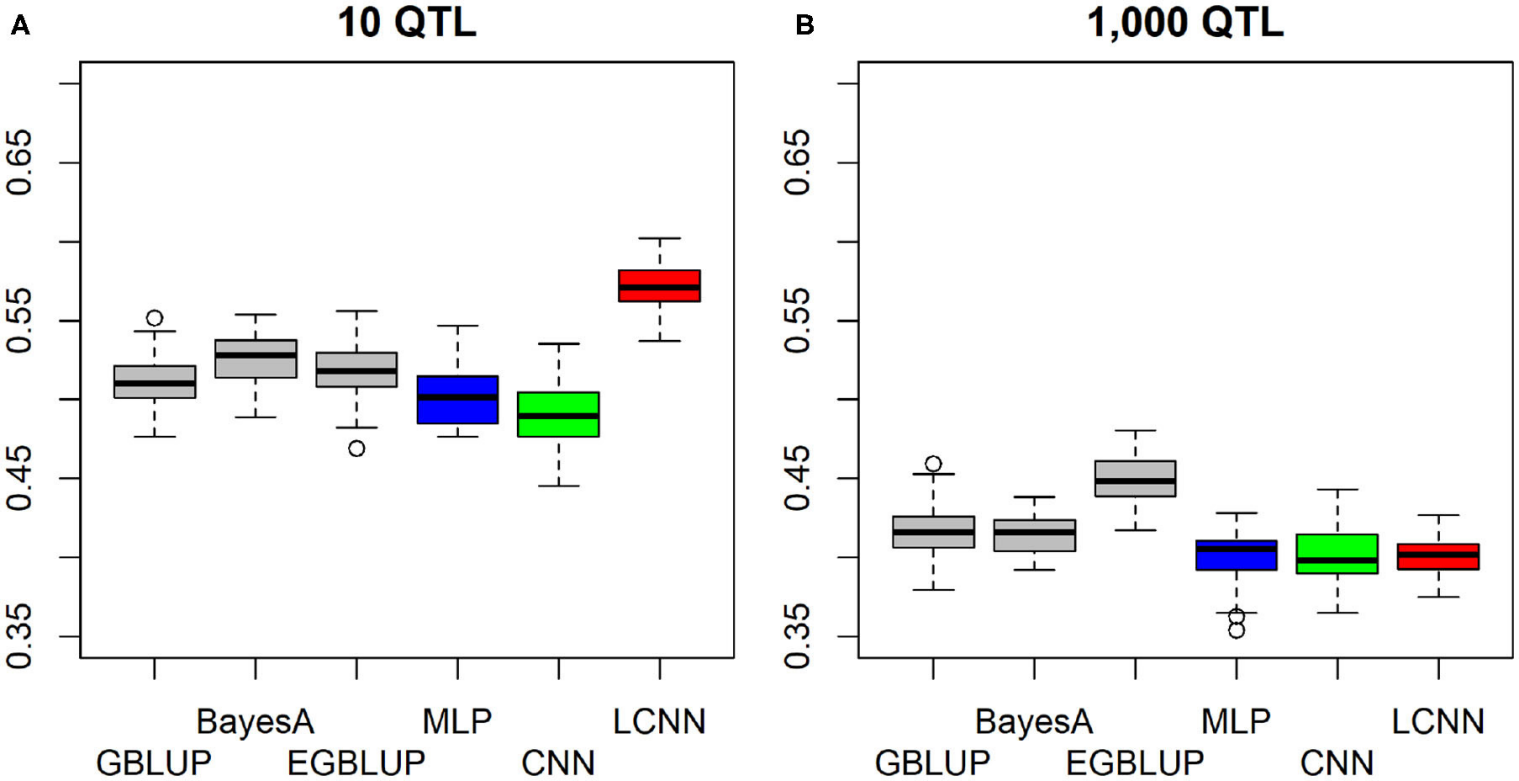
Predictive ability of different methods for genomic prediction for a simulated trait with 10 **(A)** and 1,000 **(B)** purely non-linked epistatic QTLs and a heritability of 0.5.

When considering a purely epistatic trait architecture with 10 underlying non-linked QTLs, differences between the LCNN and the other three baseline models were also around 0.06–0.08 (Figure 3A), whereas the predictive ability in the case of 1,000 underlying QTLs was very similar for all four baseline models (Figure 3B), with the GBLUP model (0.416) leading to slightly higher predictive ability (0.01–0.02).

**Figure 4:**
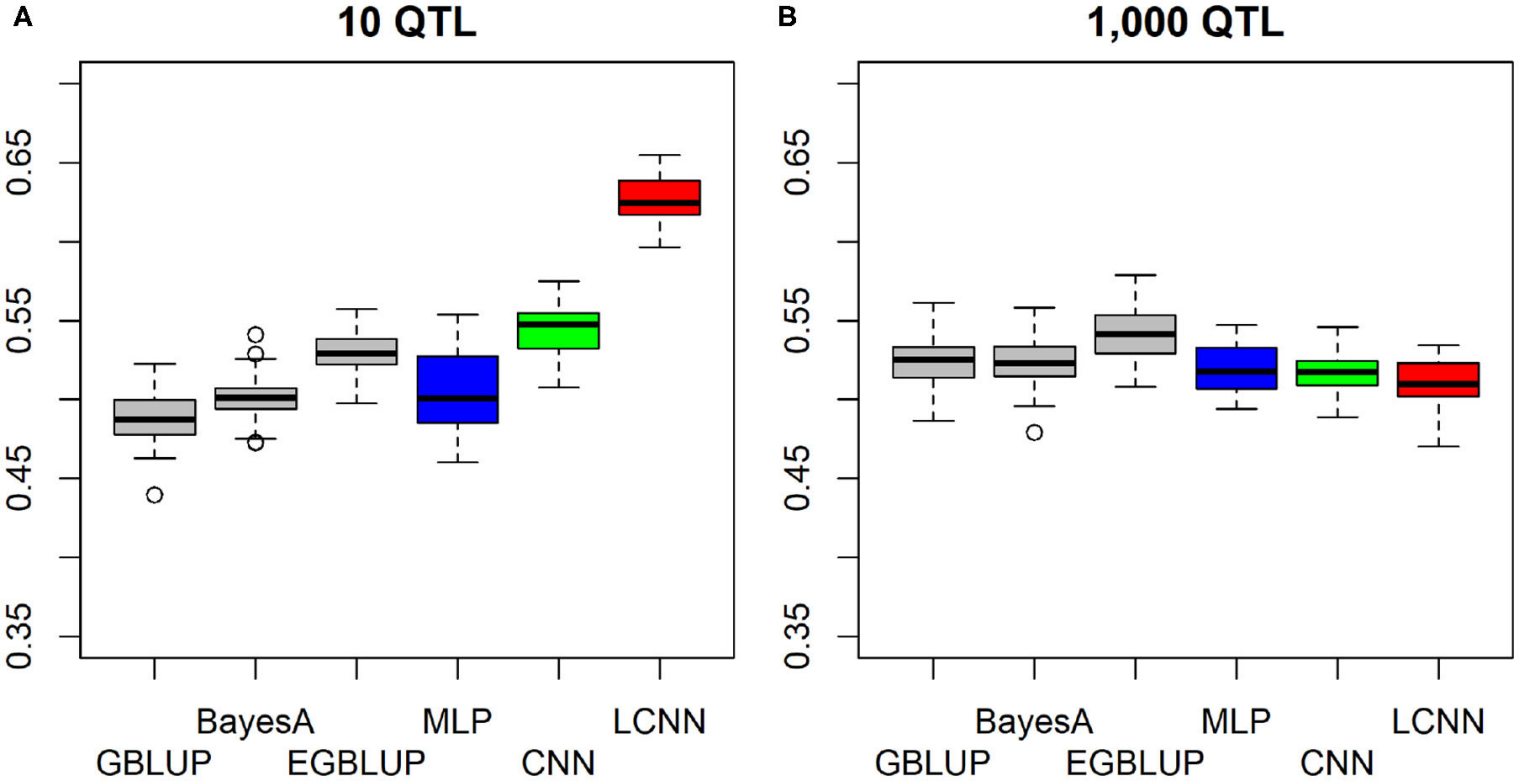
Predictive ability of different methods for genomic prediction for a simulated trait with 10 **(A)** and 1,000 **(B)** purely linked epistatic QTLs and a heritability of 0.5.

In case the underlying QTLs of the epistatic trait were placed on physically linked markers to imitate a trait caused by local interactions in a gene, both the LCNN and CNN obtained higher predictive ability when only 10 QTLs were involved in the trait, whereas the MLP and GBLUP performed worse (Figure 4A). As only CNN and LCNN are specifically accounting for local interactions, this should not be surprising. The relative differences between LCNN (0.625) and GBLUP (0.488) were here highest among all considered cases. In the case of 1,000 locally linked underlying QTLs, results of the four baseline models were again very similar with GBLUP performing about 0.01 better than the ANNs (Figure 4B). In all cases of epistatic trait architectures, the use of the EGBLUP model led to higher predictive abilities than GBLUP and was the best performing model for both traits with 1,000 underlying epistatic QTLs. For both the case of linked and non-linked traits with 10 underlying epistatic QTLs the LCNN was the best performing method.

**Figure 5:**
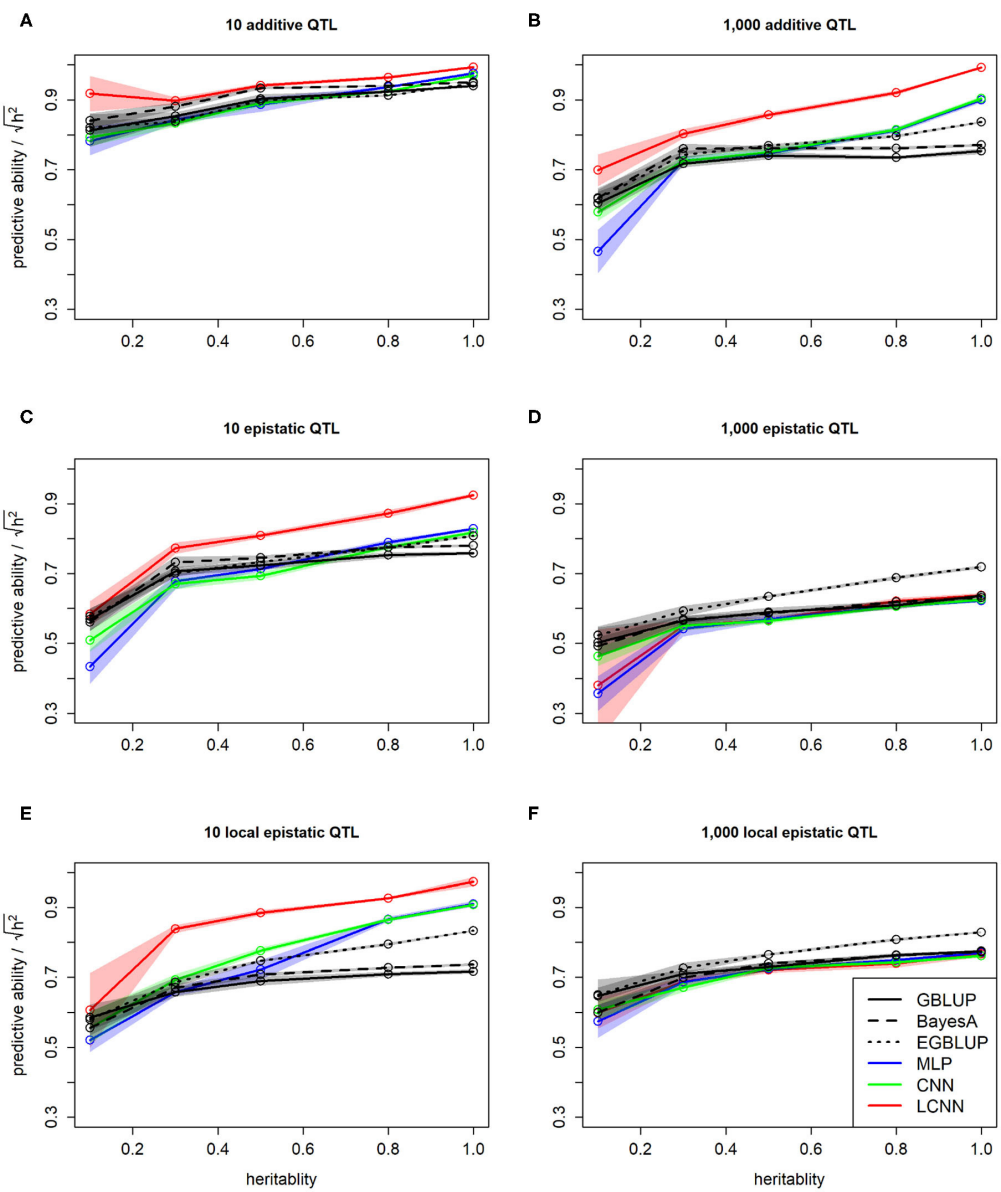
Predictive ability of different methods for genomic prediction in relation to the trait heritability for the purely additive **(A,B)**, epistatic **(C,D)**, and physically linked epistatic **(E,F)** trait with 10/1,000 underlying QTLs for the simulated maize data. Colored areas indicate 95% confidence intervals for the mean value.

As expected, we observed a higher predictive ability for traits with higher heritability. This was not only the case when comparing absolute values but also when dividing by the square root of the heritability to standardize performances. This standardization was applied as this is the highest theoretical achievable predictive ability for a given heritability (Figure 5). Overall, obtained standardized predictive abilities for the additive traits are higher and close to the maximum in the case of 10 additive underlying QTLs (Figure 5A). In particular for cases of high heritability, the LCNN is outperforming all other models for the additive trait with 1,000 QTLs and the two epistatic traits with 10 QTLs (Figures 5B,C,E). For the epistatic traits with 1,000 QTLs all models performed on a similar level, with CNN/LCNN performing worse for cases of low heritability (Figures 5D,F).

**Figure 6:**
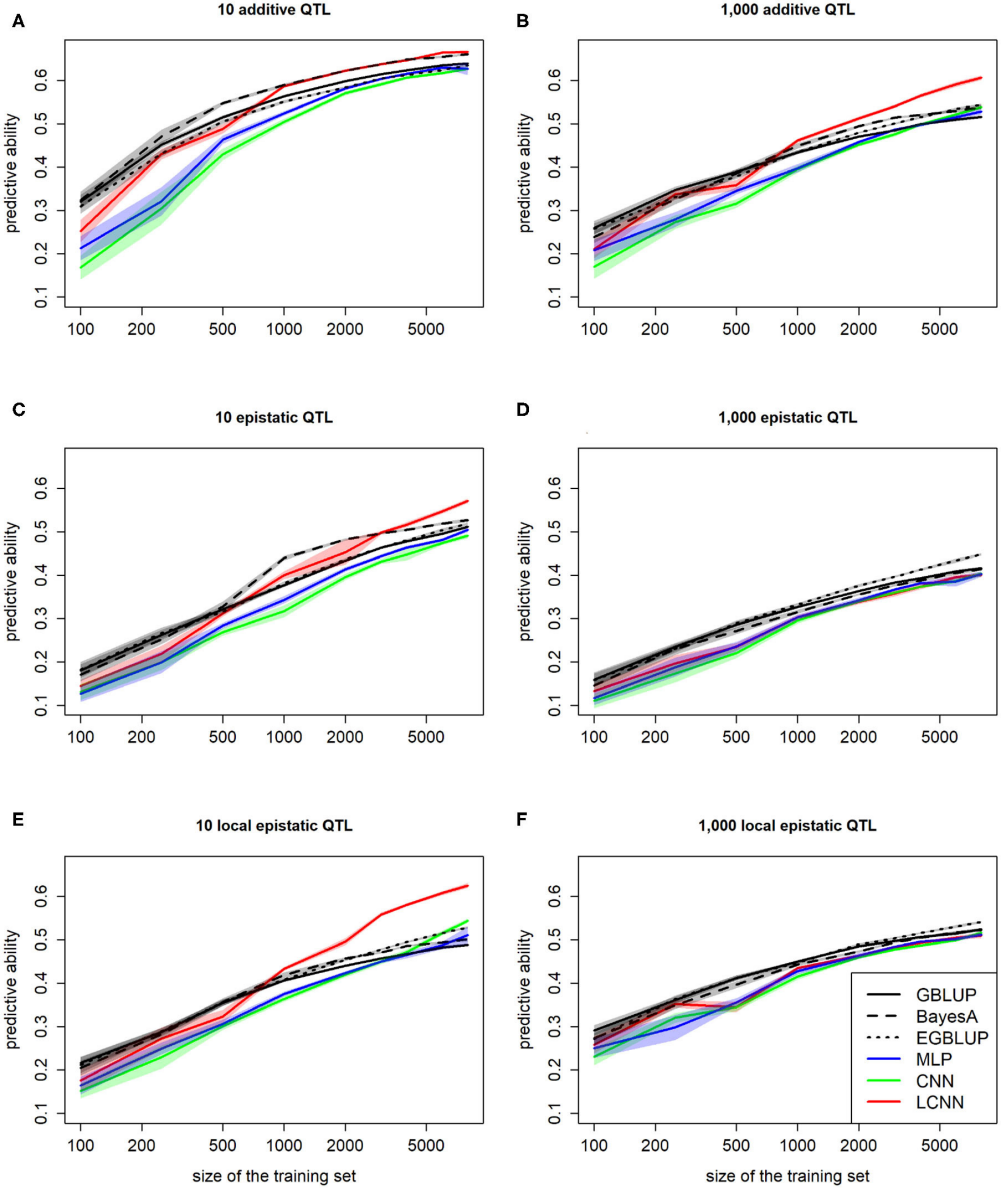
Predictive ability of different methods for genomic prediction depending on the size of the training set for purely additive **(A,B)**, epistatic **(C,D)**, and physically linked epistatic **(E,F)** trait with 10/1,000 underlying QTLs and a heritability of 0.5. Colored areas indicate 95% confidence intervals for the mean value.

When comparing the predictive ability depending on the number of individuals used for model fitting, we observed an inferior performance of all three classes of ANN models relative to GBLUP for small training sets (100, 250; Figure 6). However, of the three ANN classes considered, the LCNN is still performed best. In particular for traits with 1,000 purely additive QTLs and 10 epistatic QTLs, the LCNN outperformed the GBLUP model when considering a training panel with 1,000 or more individuals. Overall, we observed higher gains in predictive ability in the ANNs compared to the considered LMs. As ANNs are known to be extremely data hungry (Goodfellow et al., 2016) this should not be that surprising, but it also shows the promise of the method for large scale data sets. Overall, the network architectures with less layers and parameters were less affected by the reduced size of the training set.

### 3.3. Comparison Between LCNN Models

**Figure 7:**
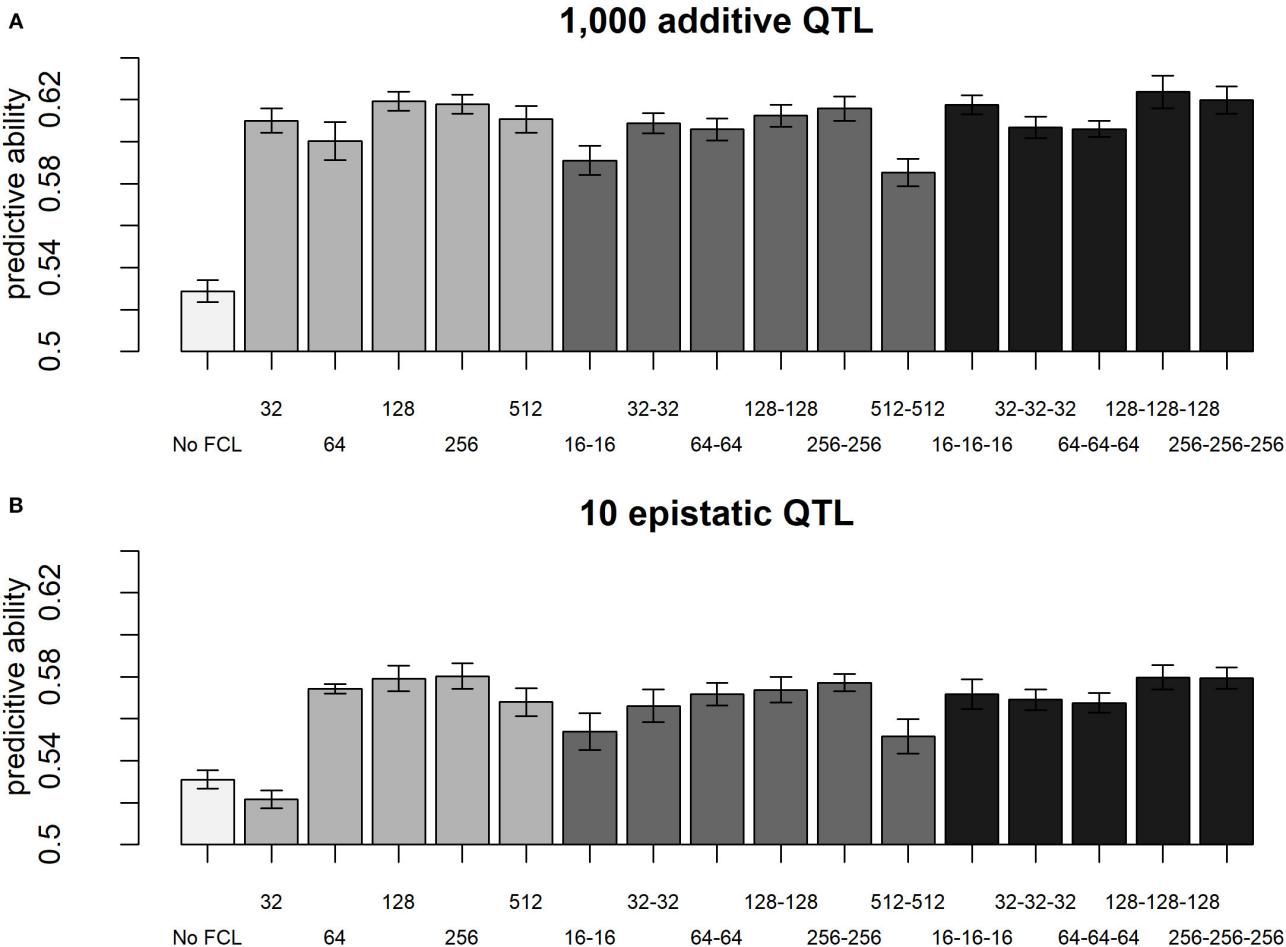
Predictive ability of different layer designs of the LCNN with modifications to the FCLs for the purely additive trait with 1,000 QTLs **(A)** and the epistatic trait with 10 QTLs **(B)** and a heritability of 0.5.

When comparing different layer designs for the LCNNs, we observed small, but still statistically significant differences between the different network architectures. In particular for purely additive traits, larger kernel sizes (KS) in the LCL led to higher accuracies (KS 5: 0.603; KS 10: 0.606; KS 20: 0.616; KS 40: 0.618—two-sample *t*-test: *p*-value < 0.0001 for KS 5 against KS 40), whereas the stride had neglectable impact (Supplementary Figure 1). In regard to the design of the subsequently applied FCLs, the performance for most models was similar, unless the design was extremely small (e.g., one layer 32 nodes) or extremely large (e.g., two layers with 512 nodes; Figure 7). A potential reason for this is over/underparametrization in the applied network architectures. Best results were obtained in network architectures with a relatively high number of nodes per layer (128/256). With more individuals used for model fitting, we would expect larger network architectures to perform better (Goodfellow et al., 2016). Although, differences between network architectures are significantly different, we would still argue that they are too small and trait specific to incline real practical relevance that would justify extended cross-validation schemes to optimize.

### 3.4. Multi-Trait Models

**Figure 8:**
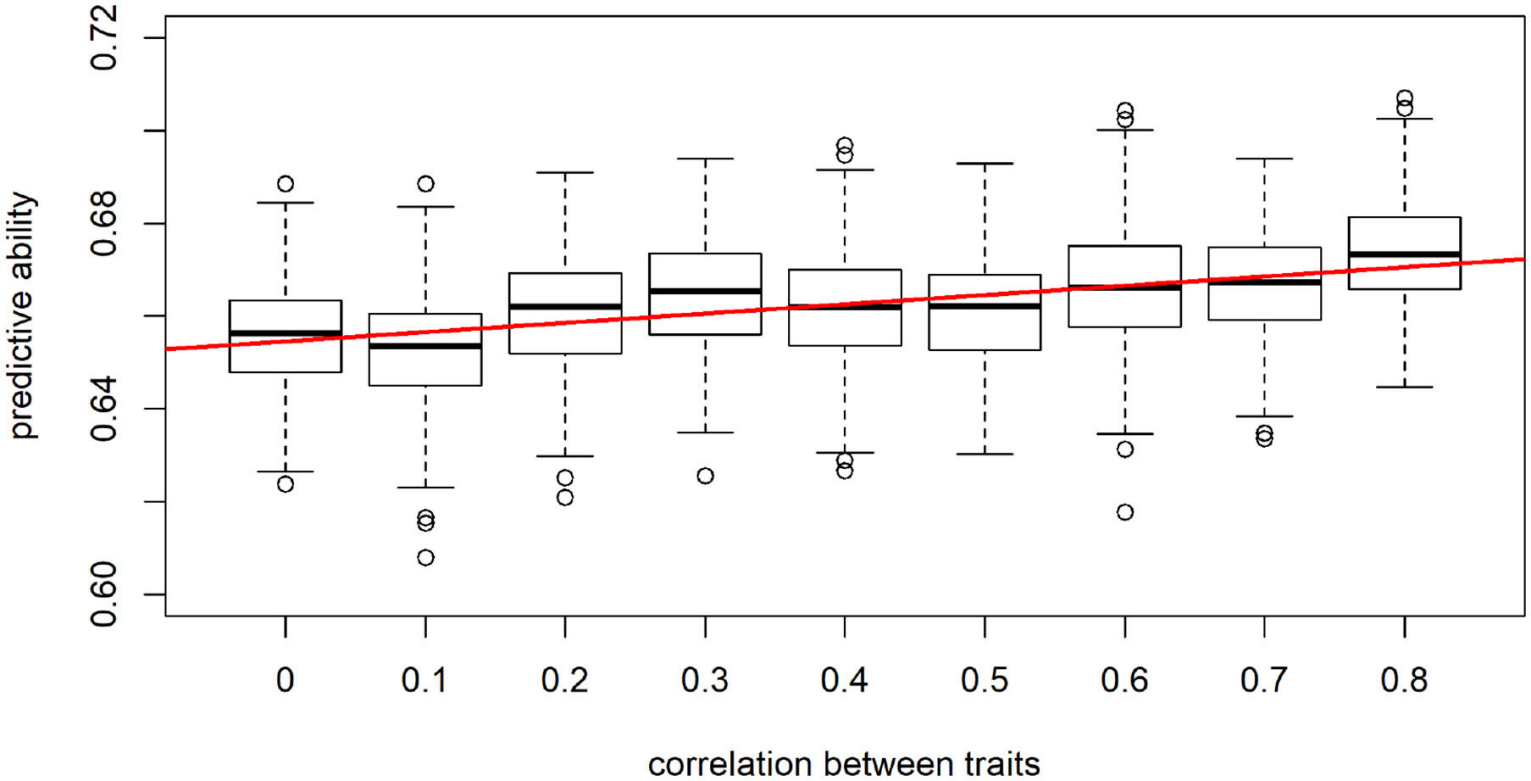
Predictive ability of the multi-trait LCNN models dependent on the correlation between traits.

When considering a data panel of 8,000 individuals and 5 traits, performance of the LCNN significantly increased with trait correlation (*t*-test: *p*-value = 0.0013, Figure 8). For example, for uncorrelated traits an average predictive ability of 0.656 compared to 0.672 for highly correlated traits (0.8) was observed. Although this difference might look small, one needs to consider here that the maximal obtainable predictive ability for a trait with heritability of 0.5 is 0.707.

As computing times of the GBLUP model were extremely high when using ASRemlR (Butler et al., 2009), only reduced data panels with two traits and limited numbers of individuals were tested. However, difference between models and traits were minor with no systematic gains for the single or multi-trait models. Note that far higher gains in a mixed model should be expected when considering partially available phenotypes. In an ANN this could be modeled by including the available phenotypes as an additional input, but again requires uniform inputs from all individuals.

### 3.5. Arabidopsis Data

**Figure 9:**
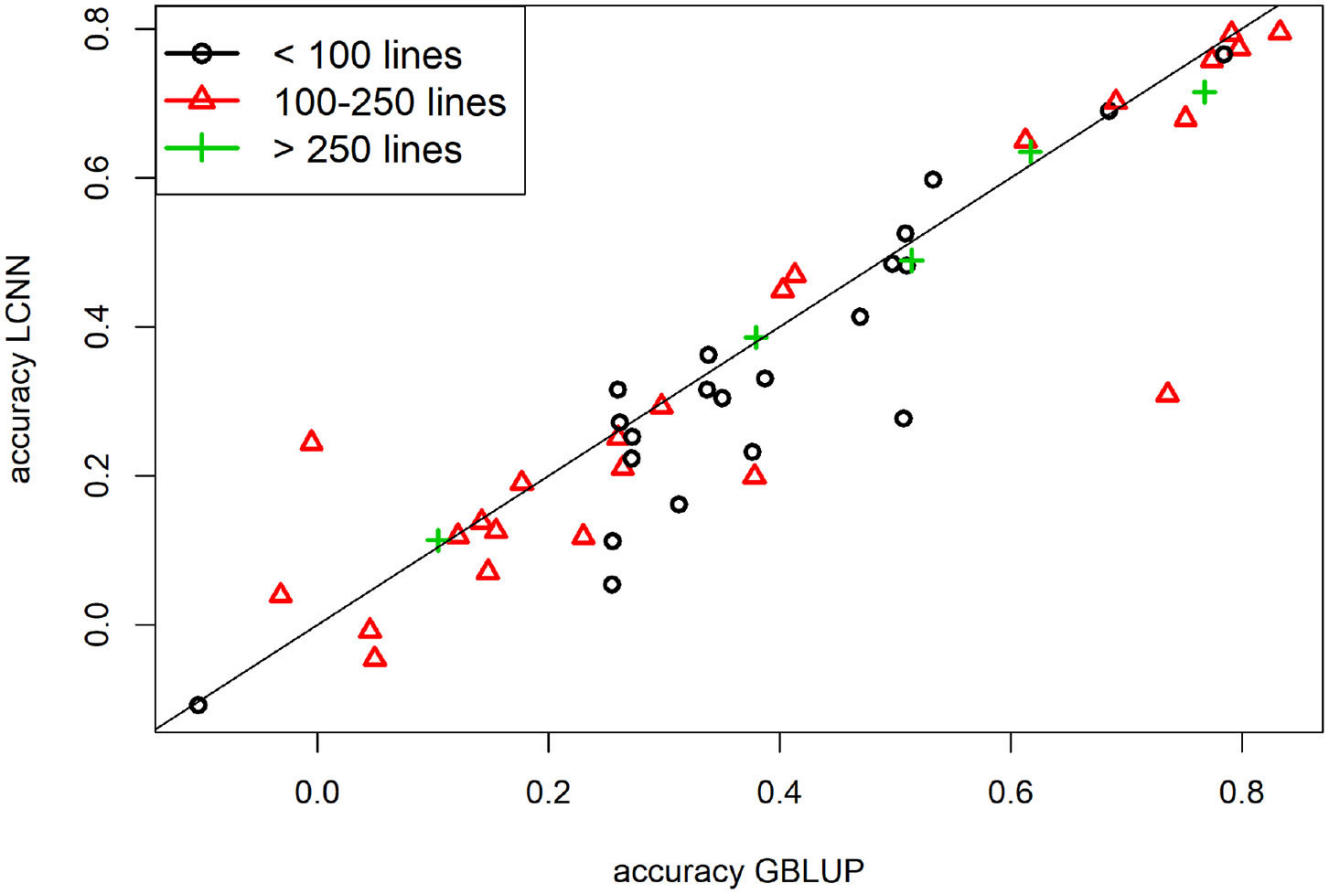
Predictive ability for GBLUP and the LCNN model for the different Arabidopsis traits in relation to the size of the training set and no validation set.

When comparing the model performance for the different ANNs for the Arabidopsis data set, the highest average predictive ability was observed for the LCNN model (0.340) compared to 0.316 for the MLP and 0.312 for the CNN model (Table 1). However, all three ANNs were outperformed by the considered models from the LM class. The differences between the ANNs and LMs decreased for traits with higher number of individuals used for model fitting. Whereas differences for traits with <100 individuals on average were 0.078 between GBLUP and the LCNN, this difference is reduced to 0.037/0.021 for traits with more than 100/250 lines in the training set (Table 1). The variance in obtained predictive ability was highest for MLP (0.031) and CNN (0.031) compared to the LCNN (0.029) and lowest for the linear models (0.024). Note that no traits with more than 468 phenotyped lines were considered here and gains in the simulated data were typically only obtained when using at least 1,000 lines (Figure 6). When using a set number of epoch and no validation set the overall accuracies increased for all three considered ANN architectures and differences in predictive ability between the ANNs and GBLUP halved (Table 2, Figure 9). One exception to this is the trait flowering time in the field (FT_field) which resulted in extremely unstable models for all three ANNs with 20% of all trained models leading to basically zero predictive ability and 55% lower average predictive ability. To our knowledge there is no immediately obvious reason why these models should not work for this particular trait, making this issue even more critical. Details on the predictive ability of the individual traits and the number of phenotypes considered for each trait are given in Supplementary Table 4. Results reported here were obtained when using the baseline network architectures. The interested reader is referred to Freudenthal (2020) for details on extended benchmark tests with varying model architectures. Note that after trait-specific model architecture tunings in Freudenthal (2020), higher predictive ability with the LCNN compared to GBLUP were obtained for 33 of the 52 traits with *h*^2^ > 0.5, whereas only 27 of the 93 traits with *h*^2^ < 0.5 benefited from the use of a LCNN compared to GBLUP.

**Table 1:** Average predictive ability for the different models for the Arabidopsis traits in relation to the size of the training set.

**Trait architecture**	**GBLUP**	**BayesA**	**EGBLUP**	**MPL**	**CNN**	**LCNN**
Average predictive ability (all)	0.390	0.382	0.382	0.316	0.312	0.340
Average predictive ability (training set < 100)	0.385	0.367	0.376	0.297	0.291	0.313
Average predictive ability (100 < training set < 250)	0.376	0.375	0.368	0.324	0.318	0.338
Average predictive ability (training set > 250)	0.477	0.477	0.472	0.358	0.370	0.456

**Table 2:** Average predictive ability for the different models for the Arabidopsis traits in relation to the size of the training set and no validation set.

**Trait architecture**	**MPL**	**CNN**	**LCNN**
Average predictive ability (all)	0.346	0.348	0.354
Average predictive ability (training set < 100)	0.332	0.332	0.336
Average predictive ability (100 < training set < 250)	0.353	0.352	0.347
Average predictive ability (training set > 250)	0.370	0.392	0.468

### 3.6. Computing Time

Tests for the ANN models were performed using a GeForce RTX 2080 Ti graphics card and Intel(R) Xeon(R) Gold 6154 processor (3.00 GHz) with 18 cores, whereas all LMs were fitting on a server cluster with Intel E5-2650 (2X12 core 2.2 GHz).

**Figure 10:**
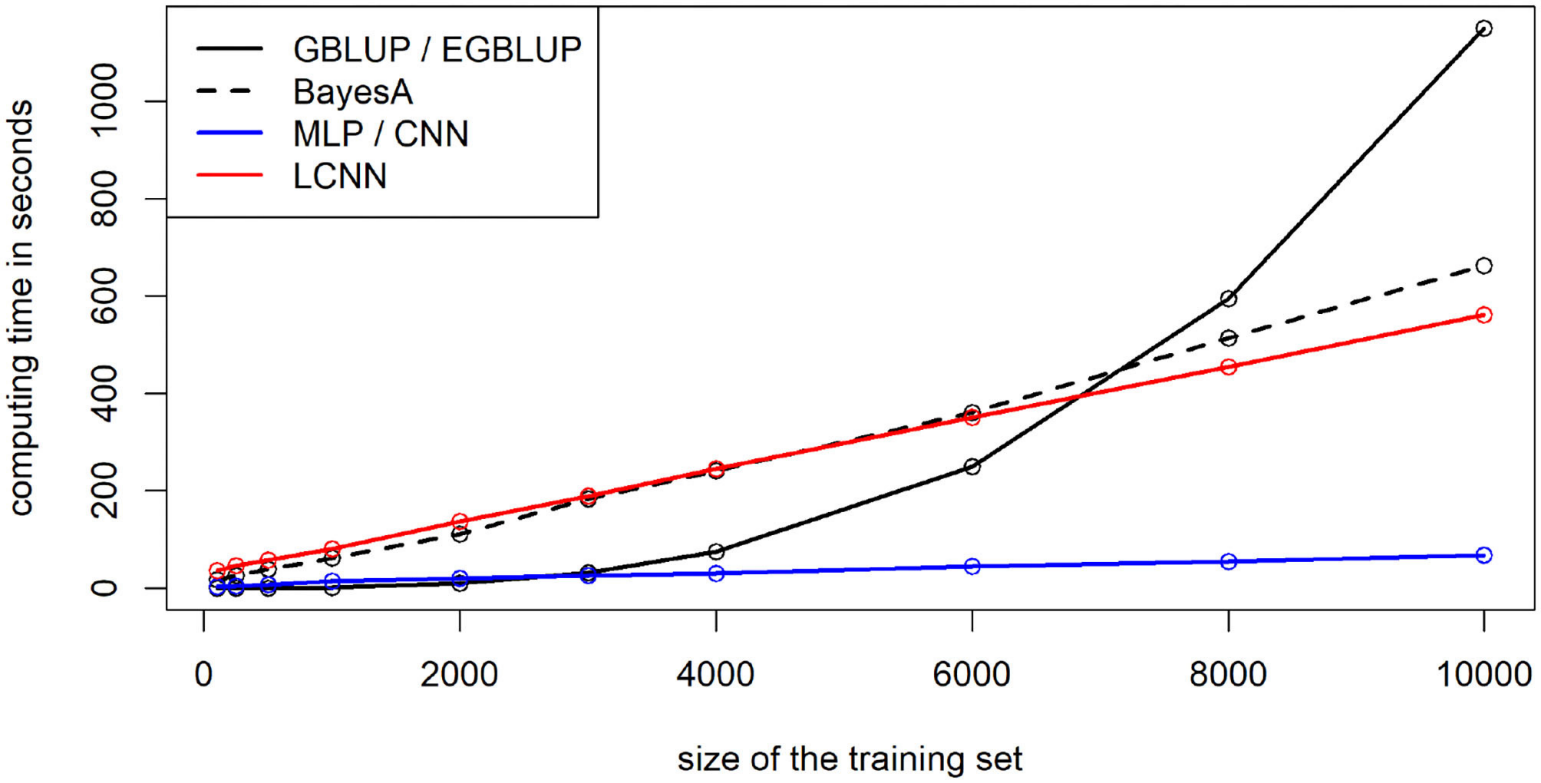
Computing time for training of the maize data depending on the number of individuals used for model training.

When using 8,000 individuals for model training, computing times for the model fitting for the MLP and CNN were lowest with 0.9 min (Figure 10), followed by the LCNN (7.6 min) with LMs taking longest (GBLUP/EGBLUP: 9.9 min, BayesA: 8.6 min). Note that differences in computing time between LM and ANN methods can also partially be attributed to faster computing resources, as a high-end graphics card was used for the training of the ANNs. However, this will only impact the absolute computing time and not the scaling in the number of individuals. Computing time increased only linearly for the considered ANNs, whereas a cubic increase in the number of phenotyped individuals was observed for the GBLUP model (quadratic scaling in the total number of individuals). In all ANNs 50 epochs were performed, although the finally chosen model was usually obtained within the first 10 training epochs. As the number of required epochs should decrease with larger training populations one could even argue that ANNs have a less than linear scaling. Scaling for the Bayesian methods was linear in both the number of phenotyped and non-phenotyped individuals. In terms of memory requirements the ANN methods at peak required 14 GB, whereas GBLUP/BayesA used up to 22/24 GB. In terms of scaling, the ANNs should again be favorable in large scale data sets, as ANNs have a linear scaling in terms of memory, whereas all considered LMs have an approximately quadratic scaling in the number of individuals considered.

Multi-trait models in ASRemlR (Butler et al., 2009) required extreme computational load with even a two trait model with 5,000 individuals requiring 400 GB memory and 34 h of run-time. Consider here, that for practical applications the computational load in mixed models is reduced by using approximations, fixing variance components and only considering bi-variate models. The five trait model in the three considered ANN architectures took basically the same time as the fitting of one single trait model (MLP/CNN: 0.9 min, LCNN: 7.7 min).

## 4. Discussion

A common misconception of ANNs is that they are treated as black boxes, causing back propagation of causal variants and fundamental design questions to be second-order problems. Note that the baseline MLP model used for the simulated maize dataset resulted in a model with 2.2 million interdependent parameters. As “only” 8,000 individuals were used for training, this can potentially lead to massive overparametrization issues (Fan et al., 2014). The use of a CL is reducing this problems substantially with our baseline CNN “only” needing 225,610 parameters. Potential problem with this is that, filters in a CLs assign effects to specific sequences of input variants. However, adjacent markers on a SNP-array usually have no underlying direct functional relation (e.g., protein coding). Thus, the same sequence of adjacent marker variants in different areas of the genome should not have any related effects. Therefore, this modeling approach does not really seem appropriate from a genetics perspective and potentially makes it more difficult to obtain a good model fit. The newly proposed LCNN fixes these issues by introducing region-specific filters. This increases the number of required parameters in the model slightly (260,195), but still is a massive improvement in terms of number of parameters compared to the MLP.

On the contrary, when working with whole genome sequences on the level of single genes the use of CNNs has shown to be quite useful (Washburn et al., 2019). However, using whole-genome sequence data does not reflect the currently available standard for genomic prediction, as no significant gains in most applications are reported when using more than just low (~10 k SNPs) to medium (~50 k SNPs) density SNP arrays (Ober et al., 2012; Erbe et al., 2013), generating such sequence data is still costly (Schwarze et al., 2018) and problems of even higher overparametrization can arise here.

As shown by the results above, the use of a LCNN can substantially improve the accuracy of genomic prediction compared to more frequently applied ANNs architectures like MLPs and CNNs for both simulated and real data sets, and independent of the size of the training set. However, compared to state-of-the-art methods like GBLUP, results in both simulated and real data for smaller training populations (100–500) showed substantially worse performance, both in terms of predictive ability and model robustness, of all considered ANN architectures. On the contrary, for large scale data set with up to 8,000 individuals used for training, the LCNN is outperforming GBLUP, or at least performing on a similar level, for basically all simulated trait architectures. As results for the simulated and real data were very similar for smaller training populations, we would expect results to generalize to large scale real data sets. However, as typical data panels in plant breeding contain <1,000 lines, we must conclude that as of today, genomic data sets in plant breeding are usually too small to justify the use of ANNs in breeding application.

On the contrary, even larger populations with potentially millions of animals are available in livestock breeding (Masuda et al., 2016). However, the obtained predictive ability should not be the only factor to consider when deciding which model to use in practice. For example, consider that ANNs require an unified input from all considered individuals, but in animal breeding the use of single step GBLUP (Legarra et al., 2009; Aguilar et al., 2010; Christensen and Lund, 2010) to perform a joined breeding value estimation for genotyped and non-genotyped animals via combination of a genomic and a pedigree based relationship matrix is common practice. Furthermore, there are no direct counterparts to the estimation of both heritability and reliability, which can be required in subsequent steps (e.g., construction of selection indices; Hazel and Lush, 1942; Miesenberger, 1997). Depending on the application, there are potential solutions to this like the estimation of the heritability via repeated measurements, pedigree-based approaches for the estimation or numerical approximation. Another aspect to bear in mind is that breeding values are additive by design, so it is not even clear if a higher predictive ability will actually result in higher genetic gain (Martini, 2017).

On the positive side, ANNs also come with great potential. Most prominently, models are computationally extremely efficient, with computing times only increasing linear in the number of individuals considered. As state-of-the-art methods like GBLUP have a cubic cost or require approximated solutions, we would expect great usefulness of ANNs for large scale data set. Similarly, including additional inputs like other omics (Li et al., 2019) or multi-trait models (Calus and Veerkamp, 2011; Lyra et al., 2017) is computationally extremely challenging and therefore usually neglected. In the context of ANNs, computing time by adding additional input or output layer to the models comes with no substantial increase in computing time.

Overall, we can conclude that, as of today, ANNs will not be the ideal model in most current breeding applications unless uniform genomic data for all relevant individuals are available. However, with reduced genotyping costs, advances in high-throughput phenotyping and additional inputs and outputs to consider, this might change in the future. Of the tested ANN structures, we see the most potential in the newly proposed LCNN architectures, as negative aspects like the overparametrization in MLPs and filters not being in line with our prior knowledge of genetic traits for CNNs are avoided. This is also empirically supported by the conducted test for both the simulated and real data sets as obtained predictive abilities are increased for basically all considered traits when using a LCNN architecture. Therefore, LCNNs should be a key network architecture to consider when designing artificial neural networks for the use in genomic prediction, or even in general when performing practical applications that are using genomic data from a genotyping array as an input.

## Data Availability Statement

Publicly available datasets were analyzed in this study. This data can be found at: https://arapheno.1001genomes.org/; https://link.springer.com/article/10.1007/s00122-019-03428-8.

## Author Contributions

TP lead the development of the methodology, performed the analysis, and wrote the initial manuscript. JF, AK, and HS provided critical feedback to both analysis and the manuscript. HS supervised the study. All authors read and approach the final manuscript.

## Conflict of Interest

The authors declare that the research was conducted in the absence of any commercial or financial relationships that could be construed as a potential conflict of interest.
