# Supplementary figures and images for: Using Local Convolutional Neural Networks for Genomic Prediction
Source: Front Genet. 2020 Nov 12;11:561497. doi: 10.3389/fgene.2020.561497 (PMC7689358; doi:10.3389/fgene.2020.561497)

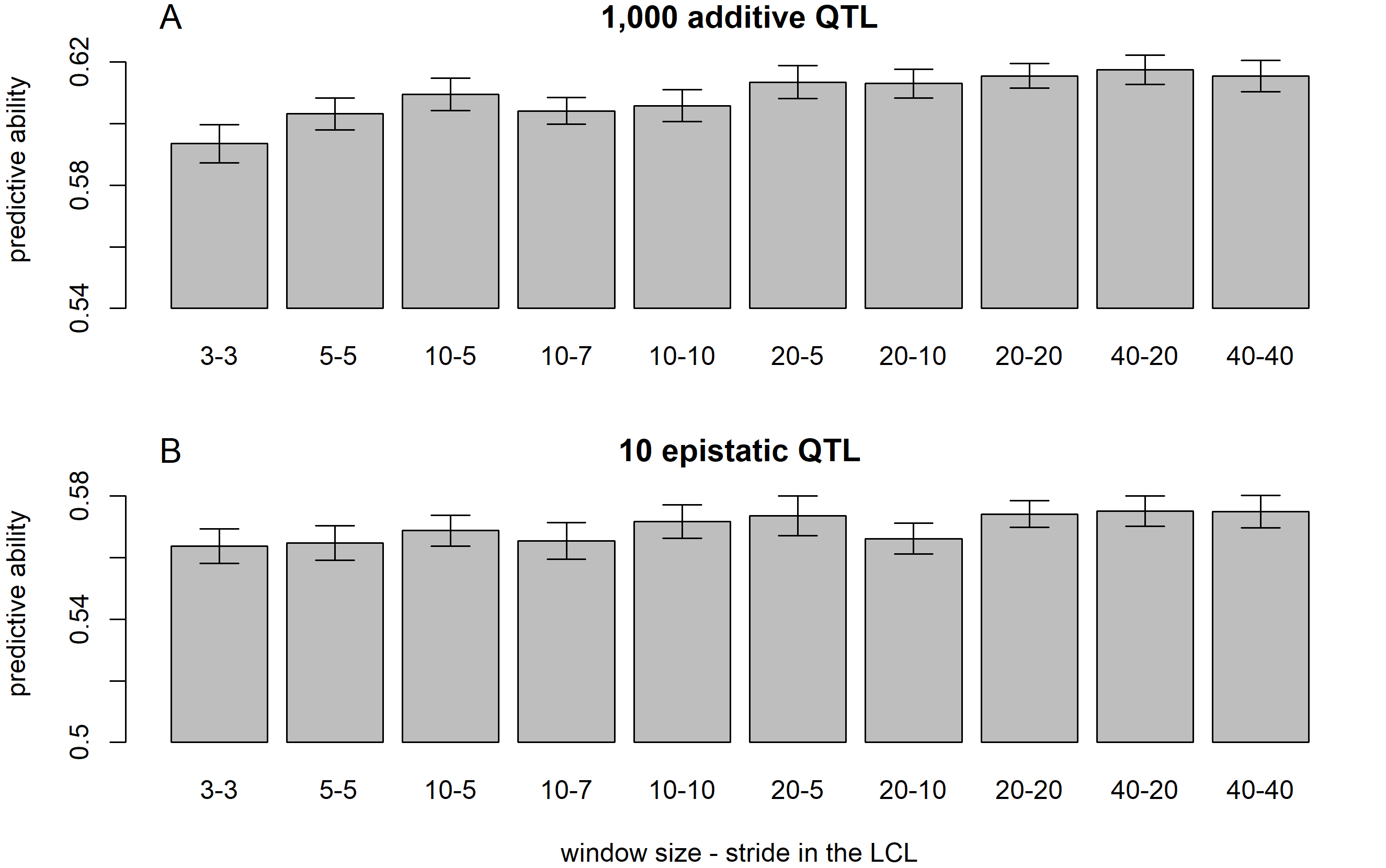

Supplement: Supplementary file 8 [file Image_1.png]
